# 

**Published:** 1851-06

**Authors:** 


No. I. JUNE. Vol. VII.

### BUFFALO MEDICAL JOURNAL AND MONTHLY REVIEW OF MEDICAL AND SURGICAL SCIENCE.

EDITED BY AUSTIN FLINT, M. D.

BUFFALO:
PUBLISHED BY JEWETT, THOMAS & CO.
Commercial Advertiser Buildings.
1851

